# ACE/ACE2 balance might be instrumental to explain the certain comorbidities leading to severe COVID-19 cases

**DOI:** 10.1042/BSR20202014

**Published:** 2021-02-01

**Authors:** Sarbashri Bank, Subrata Kumar De, Biswabandhu Bankura, Smarajit Maiti, Madhusudan Das, Gausal A Khan

**Affiliations:** 1 Department of Biochemistry, Vidyasagar University, Medinipur, India; 2 Cardiovascular disease section, Sinha Institute of Medical Science and Technology, Kolkata, India; 3 Department of Zoology, Vidyasagar University, Medinipur, India; 4 Department of Zoology, University of Calcutta, Kolkata, India; 5 OIST, Biochemistry and Biotechnology, Medinipur, India; 6 Department of Physiology and Physiotherapy, CMNHS, Fiji School of Medicine, Fiji National University, Fiji

**Keywords:** ACE/ACE2, Comorbidity, Hypoxia, SARS-CoV-2

## Abstract

The outbreak of Severe Acute Respiratory Syndrome Coronavirus-2 (SARS-CoV-2) is a global catastrophe. The elderly and people with comorbidity are facing a serious complication of the disease. The entry and infection strategy of SARS-CoV-2 in a host cell is raised by an amazing way of angiotensin-converting enzyme (ACE) 2 (ACE2) receptor recognition and imbalance of ACE/ACE2 in various organs, especially in the lungs. Here it has been discussed the role of interferon and protease during the receptor recognition (begining of infection) and followed by the impact of cytokine and hypoxia in the context of the balance of ACE/ACE2. It has also very concisely delineated the biochemistry and mechanism of ACE/ACE2 balance in different stages of infection and its role in comorbidity.

Severe Acute Respiratory Syndrome Coronavirus-2 (SARS-CoV-2) disease is an emerging global threat. Old age or people with any age who have serious chronic health issues (non-monitored hypertension, heart disease, obesity, diabetes, cancer, immuno-suppression status) are more susceptible to the complication and severity of the disease (https://www.cdc.gov/coronavirus/2019-ncov/need-extra-precautions/index.html).

## Entry of SARS-CoV-2 and its infection

### Interferon stimulation

**Figure 1:**
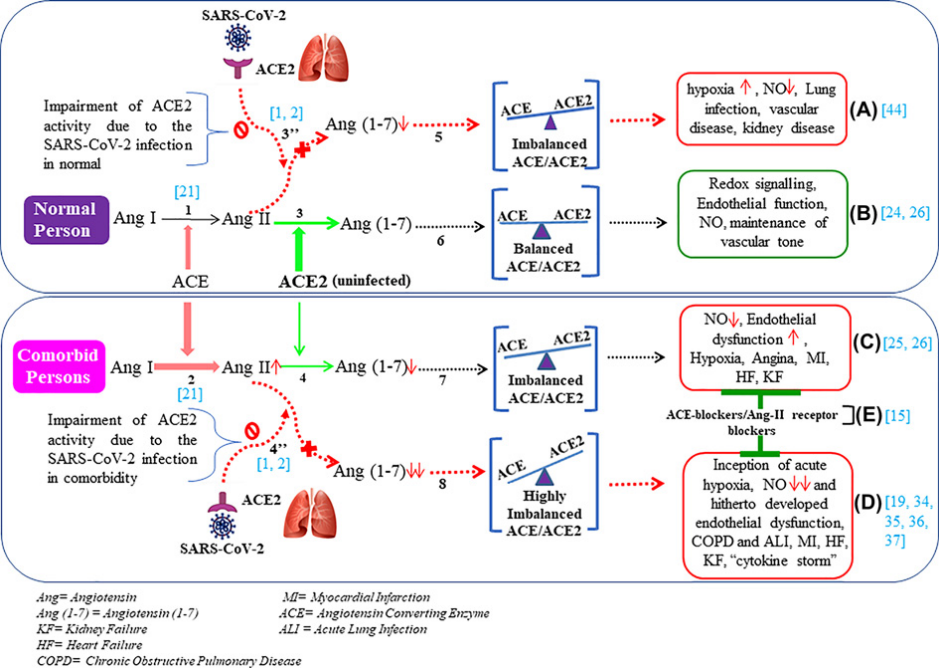
Balance of ACE and ACE2 activity in normal individuals and hypertensive/hyperglycemic persons and in case of infection by SARS-CoV-2 ACE converts Ang I into Ang II (arrow 1 and 2; where thick reddish arrow-(2) indicates the higher activity of ACE in hypertensive/hyperglycemic/CKD, i.e. comorbid patients) and ACE2 converts Ang II into Ang (1-7) (arrow 3 and 4) (thick green arrow demonstrates higher ACE2 activity in normal) and dotted red arrows [3” and 4”] indicate ACE2 activity (impaired activity Ɵ) after SARS-CoV-2 binding. In normal, ACE activity is counterbalanced by ACE2, (arrow 6) i.e. ACE≈ACE2; but in diseased state, imbalance of ACE and ACE2 (indicates arrow 7) and further more while SARS-CoV-2 binds to ACE2, there is high imbalance of ACE and ACE2 activity, i.e. ACE >> ACE2 because of impairement of ACE2 (arrow 8). When normals are infected by SARS-CoV-2, the balance of ACE and ACE2 shows in arrow 5. Boxes A–D represent the effect of imbalace of ACE and ACE2 activity. And this regulatory mechanism (balance/imbalance) of ACE and ACE2 is global in certain cell types. Turqoise bcakets ([ ]) represent related references in the figure.

It has been found that SARS-CoV-2 spike protein (S1) binds to its receptor, angiotensin-converting enzyme (ACE) 2 (ACE-2) (Figure 1: 3” and 4”) to enter human lung cells (bronchial ciliated epithelial cell and type II pneumocytes) similar to the action of SARS-CoV [1,2]. Induction of the interferon-stimulated gene (ISG) is significant for the antiviral defense mechanism [3,4]. ACE2 (STAT1-binding sites) has been reported as an ISG in epithelial cells [5]. The fallacy is that, SARS-CoV-2 recognizes ACE2 to enter host cells, so SARS-CoV-2 could utilize the ACE2-mediated tissue-protective response to provide additional cellular entry targets. This scheme employed by SARS-CoV-2 could pose serious threat to the human host [6]. The balanced role of IFN (type I, II and III) in tissue protection and host restriction of SARS-CoV-2 infection is, therefore, significant [5,7].

### Host protease and bonding with receptor

Host cell proteases (cathepsin, trypsin factor X, furin and TMPRSS2) impart a major role in the priming of viral spikes and their entry into the cell via receptor binding [7]. Both spike proteins and ACE2 are proteolytically modified during bonding and entry. The binding affinity of SARS-CoV-2 to ACE2 is stronger than SARS-CoV, with modifications in several amino acid residues [8] that lead to augmented hydrophobic interactions and salt bridge structures [9,10]. This may explain the significantly greater infectivity and spreading ability of COVID-19 than the previously occurring SARS. ADAM-17, a disintegrin and metalloproteinase 17, has a proteolytic effect on ACE2 [11,12]. It was also found that over-activated renin–angiotensin system (RAS) can enhance ACE2 shedding and subsequently the up-regulation of ADAM-17 (increase in ADAM-17 activity due to the ROS-induced phosphorylation [13]) induces heart failure, acute coronary disease due to the loss of ACE2. As such Ang II is accumulated and impairment of conversion of Ang II into Ang (1-7) leads to RAS-mediated pernicious effect in a feedback cycle [14,15]. It could be possible that S1 protein-bound ACE2 receptors may not function properly and that the adverse interaction between SARS-CoV-2 and ACE2 may be more prominent in males due to the androgenic hormone testosterone [16]. Action of this hormone results in the inhibition of Ang (1-7)-induced NO signaling through the angiotensin II type-2 receptor (AT_2_R) down-regulation [16]; contrarily, estrogen in females may protect this flow of adverse effects [17].

### Cytokine induction

Loss of balance between anti-inflammatory and pro-inflammatory cytokines causes chronic and mild inflammation, i.e. ‘inflame aging’ in elderly patients. This is one of the causes of diabetes mellitus [18]. Previously developed ‘inflame aging’ generates ‘cytokine storm’ during SARS-CoV-2 infection by enhanced production of TNF-α, IL-1β and IL-6 [19]. These elevated levels of pro-inflammatory cytokines are attributed to diabetes [18]. These cytokines generate adverse immunological responses during SARS-CoV-2 infection [20].

## ACE and ACE2 balance in COVID patients and in normal people

### ACE and ACE2 activity

ACE catalyzes the conversion of Ang I into Ang II [21] (Figure 1: 1 and 2); the Ang II is a vasoconstrictor that induces oxidative stress and hypertension [22,23]. On the other hand, ACE activity is counterbalanced by ACE2 which converts Ang II into Ang (1-7) which is a vasodilator (Figure 1: 3,6,B) [24]. In hypertensive or diabetic persons, the hyperactivity of ACE results in more production of Ang II than to the production of Ang (1-7) from Ang II by ACE2. As a result, it persuades endothelial dysfunction, NO inhibition, hypoxia, angina, platelet aggregation [25], myocardial infarction and kidney disorders (Figure 1: 4,7,C) [26].

### Comorbidity and SARS-CoV-2

When these comorbid patients are infected with the SARS-CoV-2 by the ACE2 receptor-recognition (Figure 1:4”), they become vulnerable. Because, previously developed abnormal endothelial cells (glycocalyx coat) become the basement of the adhesion and aggregation of large number of platelets [27,28]. In diabetes, the bioavailability of diminished NO coupling with up-regulated NADPH oxidase might be the cause of endothelial dysfunction [29]. On the other hand, it has been found that ACE2/Ang (1-7) has a significant role in the protection of CD34^+^ or CAC cell (vasoreparative function) dysfunction in diabetes [30]. So, diabetes-induced loss of CAC or CD34^+^ (vascular reparative cell) may have a role in the creation of vascular dysfunction in COVID patients and as such viral myocarditis, heart failure and cardiac arrest have been found in the patients. [30,31]. In diabetes, dysfunction of RAS is connected to heart failure with preserved ejection fraction (HFpEF) and loss of ACE2 also dampens the HFpEF. ACE2/Ang (1-7) may have significant role in the regulation of cardio-fibroblasts, cardiomyocytes and endothelial cells in case of both HFpEF and heart failure with reduced ejection fraction [32]. The ultimate eventuality is the multiple organ failure and death of the patients [33].

Patients with COPD/ARDS also face severe breathing problems due to acute lung injury (ALI) by chronic hypoxia and drastic NO-level drop (Figure 1:8,D) [34,35]. As such, the production of Ang II is so high due to high ACE activity, ACE2 is unable to counterbalance the production of Ang (1-7) (Figure 1:8). As a consequence, Ang II-induced dampening is higher in the lungs, heart and kidney cells [36,37]. It has been reported in normal individuals that the ACE2–Ang (1-7)–Mas axis imparts in the redox signaling and maintains vascular tone through the NO-induced vasodilatory action (Figure 1:B) [26] and deficiency of ACE2 might up-regulate the endothelial dysfunction and pro-inflammatory stimuli [35]. It is also significant that the oxidative stress induced by Ang II is responsible for the reduction in tetrahydrobiopterin synthesis and the uncoupling of eNOS resulting in a massive drop of endothelial NO level [38]. Vasoconstriction occurs in the increasing state of ACE/Ang II [18,19] and it might be possible that ACE/Ang II-induced hypertension may provoke hypertension-end organ damage by suppressing endothelial nitric oxide synthase activity [39]. ACE2-mediated protection of lung injury was also investigated in *ace2*-knockout mice model [40]. It was also found that immunotargeting via anti-ACE could limit the I/R injury of lungs through the antioxidative defense mechanism of pulmonary endothelium cells [41].

### Hypoxia-inducible factor and ACE2

Hypoxia-inducible factor (HIF) 1α (HIF-1α), the master regulator of oxygen homeostasis in the cell plays the crucial role during cardiac development, coronary perfusion and angiogenesis [42]. But with aging and high-risk factors (diabetes, pre-hypertension), the function of HIF-1α may not be restored and as a result, the sustained action of HIF becomes lower in COVID-19 patients, especially who are aged and have comorbid nature (diabetic, hypertensive, COPD). Individuals with HIF polymorphism are also susceptible for this disease [43]. Long-term (chronic) hypoxia-induced HIF may up-regulate ACE and down-regulate ACE2 [44]. In contrast, normal individuals who are infected by SARS-CoV-2, may face less complications than elder and comorbid patients specifically with pneumonia, ARDS, kidney injury etc. [45] and it might be due to the change of HIF level and as a consequence imbalance regulation of ACE/ACE2 ratio due to the increase in hypoxia, ACE and decrease in ACE2 imparts a major role in hypoxic pulmonary hypertension (HPH) (Figure 1:5,A) [34]. Nevertheless, in a recent study, SARS-CoV-2 infection was found occurring at a lower rate in high altitudes (>2500 m) possibly due to physiological acclimatization to hypoxia with higher HIF level and down-regulation of ACE-2 [46]. In hypoxia, the cell adapts under stress condition and HIF-1α induces the expression of pyruvate dehydrogenase kinase-1 (PDK1) which inhibits the pyruvate dehydrogenase (PDH), so pyruvate entry into TCA cycle is inhibited, as a consequence glucose metabolism shunts to glycolysis [47,48]. Thus, HIF-1α might have a fine-tuning on optimization of glucose and O_2_ utilization in hypoxic condition to produce enough energy without generating ROS through the inhibition of mitochondrial respiration [48]. The HIF-1α may play a pivotal role in chronic kidney disease by producing erythropoiesis in hypoxic conditions. Thus, the inhibition HIF-1α-hydroxylase could be a supportive therapy in sudden onset of the hypoxic condition in COVID-19 patients through the cellular adaptation [49].

### ACE and Ang II receptor blockers in COVID-19

It is reported that, ACE blockers, Ang II receptor blockers (ARBs) are supportive therapy to inhibit the Ang II-induced hypertension, kidney function and lung congestion, but these are not direct therapy against viral infection. Rather, these drugs only control the comorbid symptoms in the COVID-19 patients [50]. Few contrasting reports are also there, these ARB or ACEI augment the severity in some of the COVID patients [51]. However, it may be hypothesized that ACEI or ARB should not be withheld from the high-risk AMI, heart failure, CVD and hypertensive patients according to the statement of American Heart Association [52,53]. The targeting RAS-axes might be a way to recuperate clinical outcome of COVID patients by using ARB [15] (Figure 1:E).

## Conclusion

From the above discussion, it can be delineated that ACE2 is involved from the step of entry-infection strategy of SARS-CoV-2 to its adverse life-threatening effect in host cell (heart, lungs, kidney), so ACE/ACE2 has undoubtedly significant role in disease progression and prognosis in humans. The RAS, therefore, plays a crucial role in SARS pathogenesis and ACE2 could be a way to block the spread of SARS-CoV. ACE2 was found to be an effective therapy to control lung injury and it was also investigated in *Ace2*-knockout murine lungs [40] and further, it has been found that immunotargeting via anti-ACE could protect lungs [41]. As a result, modulation of the RAS by the balancing of ACE and ACE2 might be one of the ways to attenuate the ALI in SARS-CoV and its complicacy [35] and ACE2–Ang (1-7) axis could be a potent therapeutic window for the prevention of heart failure [32].

## Data Availability

This submission cannot comply with the data type-specific deposit requirements.

## Abbreviations

ACE: angiotensin-converting enzyme
ACEI: angiotensin-converting enzyme inhibitors
ACE2: angiotensin-converting enzyme-2
ADAM-17: a disintegrin and metalloproteinase 17
ALI: acute lung injury
AMI: acute myocardial infarction
Ang II: angiotensin II
ARB: Ang II receptor blocker
ARDS: acute respiratory distress syndrome
CAC: circulating angiogenic cell
COPD: chronic obstructive pulmonary disease
eNOS: endothelial nitric oxide synthase
HFpEF: heart failure with preserved ejection fraction
HIF: hypoxia-inducible factor
I/R: ischemia/reperfusion
ISG: interferon-stimulated gene
RAS: renin–angiotensin system
S1: spike protein
SARS-CoV-2: Severe Acute Respiratory Syndrome Coronavirus-2
TMPRSS2: Transmembrane protease serine 2
